# Cytoplasmic expression of TDP‐43 in aged mice display hippocampal sclerosis‐like degeneration and neuronal loss with reduced lifespan

**DOI:** 10.1002/alz.090943

**Published:** 2025-01-03

**Authors:** Ashley J. Anderson, Matthew B Dopler, Sanaz Arezoumandan, Damian Osei‐KanKam, Stephani A. Davis, Kaouther Ajroud, Jaclyn N Lilek, Eva Bambakadis, Rachel Shapiro, Michael A. Gitcho, Margaret E Flanagan, Nigel J Cairns

**Affiliations:** ^1^ Delaware State University, Dover, DE USA; ^2^ Delaware Center for Neuroscience Research, Dover, DE USA; ^3^ University of Pennsylvania, Philadelphia, PA USA; ^4^ UT Health San Antontio, San antonio, TX USA; ^5^ University of Texas Health San Antonio, San Antonio, TX USA; ^6^ Glenn Biggs Institute for Alzheimer’s & Neurodegenerative Diseases, University of Texas Health Sciences Center at San Antonio, San Antonio, TX USA; ^7^ Department of Pathology and Laboratory Medicine, University of Texas Health Science Center at San Antonio, San Antonio, TX USA; ^8^ Univeristy of Exeter, Exeter, MO United Kingdom

## Abstract

**Background:**

TDP‐43 is a multifunctional heterogeneous nuclear ribonucleoprotein and is the major pathological protein in motor neuron disease. Previously, TDP‐43 pathology has been described in up to 50% of those with Alzheimer’s disease. Recent evaluation of this cohort revealed a distinct pathological staging of TDP‐43 proteinopathy in an aged population, called Limbic predominant age‐related TDP‐43 encephalopathy

(LATE). Hippocampal sclerosis of aging (HS‐A) is an age‐related neuropathology characterized by severe neuronal loss and gliosis, also seen as a co‐pathology in AD and LATE. HS‐A is evident in ∼80% of cases in the hippocampal region of cases that are positive for phosphorylated TDP‐43.

**Method:**

We examined aged mice that selectively express human TDP‐43 and TDP‐43 with a defective nuclear localization signal (ΔNLS) in the hippocampus in an APP/PSEN1 background, six genotypes of interest were evaluated; WT, APP/PS1, Camk2a/TDP‐43, Camk2a/TDP‐43ΔNLS, Camk2a/TDP‐43/APP/PS1, and Camk2a/TDP‐43ΔNLS/APP/PS1 in 24 month old mice. A sample size of n = 3 was used for each genotype. Protein lysates as well as fixed tissues were prepared and analyzed.

**Result:**

These 24‐ month‐ old mice display severe neuronal loss in the hippocampus, a decrease in beta‐amyloid plaque deposition, an increase in neuroinflammation, and reduced survival.

**Conclusion:**

This aged hippocampal sclerosis‐like model may provide a greater understanding of the pathogenesis of neurodegeneration seen in TDP‐43 proteinopathies.

